# A randomised controlled trial of a self-management education program for osteoarthritis of the knee delivered by health care professionals

**DOI:** 10.1186/ar3703

**Published:** 2012-01-27

**Authors:** Sophie Coleman, N Kathryn Briffa, Graeme Carroll, Charles Inderjeeth, Nicola Cook, Jean McQuade

**Affiliations:** 1 Department of Physiotherapy, Curtin University, Beazley Avenue, Bentley, Western Australia 6102, Australia; 2 Department of Rheumatology, Fremantle Hospital, Alma Street, Fremantle, Western Australia 6160, Australia; 3 School of Medicine, University of Notre Dame, 13-19 Mouat Street, Fremantle, Western Australia 6160, Australia; 4 School of Pharmacology and Medicine, University of Western Australia, 35 Stirling Highway, Nedlands, Western Australia 6009, Australia; 5 Geriatric Medicine and Rheumatology, Sir Charles Gairdner Hospital, Verdun Street, Nedlands, Western Australia 6009, Australia; 6 Rheumatology, Royal Perth Hospital, Wellington Street, Perth, Western Australia 6001, Australia; 7 Arthritis Western Australia, 17 Lemnos Street, Shenton Park, Western Australia 6008, Australia

## Abstract

**Introduction:**

Our aim in the present study was to determine whether a disease-specific self-management program for primary care patients with osteoarthritis (OA) of the knee (the Osteoarthritis of the Knee Self-Management Program (OAK)) implemented by health care professionals would achieve and maintain clinically meaningful improvements in health-related outcomes compared with a control group.

**Methods:**

Medical practitioners referred 146 primary care patients with OA of the knee. Volunteers with coexistent inflammatory joint disease or serious comorbidities were excluded. Randomisation was to either a control group or the OAK group. The OAK group completed a 6-week self-management program. The control group had a 6-month waiting period before entering the OAK program. Assessments were taken at baseline, 8 weeks and 6 months. The primary outcomes were the results measured using the Western Ontario and McMaster Universities Arthritis Index (WOMAC) Pain and Function subscales on the Short Form 36 version 1 questionnaire (SF-36) Secondary outcomes were Visual Analogue Scale (VAS) pain, Timed Up & Go Test (TUG), knee range of motion and quadriceps and hamstring strength-isometric contraction. Responses to treatment (responders) and minimal clinically important improvements (MCIIs) were determined.

**Results:**

In the OAK group, VAS pain improved from baseline to week 8 from mean (SEM) 5.21 (0.30) to 3.65 (0.29) (*P *≤ 0.001). During this period, improvements in the OAK group compared with the control group and responses to treatment were demonstrated according to the following outcomes: WOMAC Pain, Physical Function and Total dimensions, as well as SF-36 Physical Function, Role Physical, Body Pain, Vitality and Social Functioning domains. In addition, from baseline to week 8, the proportion of MCIIs was greater among the OAK group than the control group for all outcomes. For the period between baseline and month 6, WOMAC Pain, Physical Function and Total dimensions significantly improved in the OAK group compared to the control group, as did the SF-36 Physical Function, Role Physical, Body Pain, Vitality and Social Functioning domains, as well as hamstring strength in both legs. During the same period, the TUG Test, range of motion extension and left-knee flexion improved compared with the control group, although these improvements had little clinical relevance.

**Conclusions:**

We recorded statistically significant improvements compared with a control group with regard to pain, quality of life and function for participants in the OAK program on the basis of WOMAC and SF-36 measures taken 8 weeks and 6 months from baseline.

## Introduction

Osteoarthritis (OA) is the most common form of arthritis and the third-leading cause of life-years lost to disability [1-3]. It affects 10% of the population and is more common in women than in men. By age 65 years, 50% of the population have OA [1], and, as the population ages in demographic terms, the prevalence of OA is expected to rise. The knee is a commonly affected joint, with a prevalence of OA of 40% in people age 65 years or older [2].

Self-management (SM) is considered to be an effective strategy in the treatment of chronic illnesses, including OA [4]. Numerous SM programs have been developed for different illnesses, such as diabetes, hypertension, asthma and arthritis. Various models, both disease-specific and generic, have been employed, including individual [5], group-based [6], post and internet programs [7]. Most arthritis SM programs use lay leaders who deliver a scripted program. Face-to-face interaction with health care professionals is an important component of some programs, especially when medication compliance is considered relevant [4].

A number of studies have examined the effectiveness of SM for people with arthritis [4,7,8]. One systematic review of SM interventions for various chronic diseases found a trend towards a small benefit from arthritis programs, the majority being Stanford University's Arthritis Self-Management Program (ASMP) [9] or derivatives of the ASMP. The results were not significant, and publication bias was suspected [7]. A comparison of lay leaders versus health care professional leaders has also been published [7]; however, the study required all leaders, both lay leaders and health care professionals, to deliver the same scripted program, which limited the capacity for the knowledge and skills of the health care professionals to be optimally utilised [10]. Given the design of the study, it was not surprising that no differences were demonstrated. Despite the popularity of layperson-led SM programs for arthritis, and taking into account possible cost advantages, Taylor and Bury suggested that such programs have little or no advantage in terms of improved self-efficacy or the management of chronic illness [11]. Another study, by Hurley *et al*. [5], demonstrated positive findings. They compared group and individual rehabilitation programs run by physiotherapists in combination with SM and education (ESCAPE-knee pain (Enabling Self-management and Coping with Arthritic Knee Pain through Exercise)) with normal care in 418 participants with knee OA. The authors found that people in the ESCAPE-knee pain groups had better functioning than those in the normal care group.

Although lay leaders have the potential to be role models (as they often have musculoskeletal conditions themselves), health care professionals, with their knowledge and skills, can also have powerful influence as models. A modelling approach has the potential to transmit knowledge and skills to which people may aspire, particularly if the information is perceived to be important and relevant, resulting in behaviour changes that are more likely to be maintained over the long term [12]. This platform for behaviour change is constrained in layperson-led programs because of the limited knowledge of lay leaders.

Self-efficacy is an integral component of SM, and resilience goes hand in hand with self-efficacy. Resilient people tend to have well-developed self-efficacy and, when confronted with an obstacle, will see it as a hurdle to overcome rather than an insurmountable problem. Furthermore, when repeated problems are encountered, they are more likely to persist longer in attempting to overcome them [13]. Those who attempt and succeed will benefit in terms of improved self-efficacy.

Factors influencing self-efficacy, such as problem-solving, pain management, exercise, modelling, social persuasion, weekly goal-setting and cognitive therapy, are interconnected. Pain management is important because often people are hesitant to undertake new activities for fear of pain, regardless of whether pain has previously been experienced with that particular activity. Many people with OA rely on medication for pain relief, but are reluctant to take medication because of possible side effects. Such people prefer to be aware of the pharmacologic and treatment options available and then decide on a course of management. If knowledge about the available options is lacking, however, the treatment choices are more limited, and, importantly, this may have an impact on adherence to treatment [14].

The lack of demonstrated benefits of SM in patients with arthritis highlights the need to develop and test alternative models of SM. In this report, we describe the evaluation of an SM program for people with OA of the knee, the Osteoarthritis of the Knee Self-Management Program (OAK), which is designed to be delivered by health care professionals.

People with OA may initially resist physical activity due to discomfort, fear of pain, or previous advice to avoid exercise [15,16]. Many believe that exercise will result in bone and cartilage loss and are therefore resistant to exercise in general [16], yet avoidance of activity is known to contribute to disability long term [17]. The OAK program includes general information about the benefits of exercise and specific advice on joint protection during exercise for those with OA of the knee. The program aims to maximize the benefits of physical activity and promote long-term adherence to an exercise regimen by using structured exercise participation that is linked to weekly 'SMART' goals (Specific, Measurable, Attainable, Realistic, Time-bound) [18]. The program offers the added reassurance that health care professionals are on hand to give advice, and the group dynamics offer incentives for participants to comply with and maintain the exercise regimen. Participants' success in meeting goals each week increases self-efficacy [19], which is the strongest and most consistent predictor of physical activity behaviour and its maintenance over the long term [20]. Although exercise features strongly in the OAK program, OAK is not an exercise school. Exercise is only one component of the program, and it is up to each individual to decide how much emphasis is given to exercise from week to week during the program.

Cognitive symptom management strategies are encouraged to help eliminate 'negative' symptoms associated with OA. Such negativity not only affects individual symptom control but also contributes to and exacerbates the symptoms of OA [21]. Guided imagery, relaxation techniques, positive self-talk and problem-solving are taught to participants. These techniques enable participants to understand how such influences contribute to their symptoms and provide them with the skills necessary to prevent the symptoms' becoming an overwhelming negative influence. Health care professionals also use their knowledge to assist participants with problem-solving to overcome hurdles and promote resilience [22].

## Materials and methods

In this study, we sought to determine whether a disease-specific SM program for people with osteoarthritis of the knee (the OAK program), implemented by health care professionals, would achieve and maintain clinically meaningful improvements in health-related outcomes compared with a control group. This study was approved by the Human Research Ethics Committee at Curtin University of Technology (HR141). Data access and storage were in keeping with National Health and Medical Research Council guidelines [23]. License agreements were obtained for the Short Form 36 version 1 questionnaire (SF-36) and the Western Ontario and McMaster Universities Arthritis Index questionnaire (WOMAC).

The study design adhered to Consolidated Standards of Reporting Trials Statement guidelines and intention to treat principles. This trial was registered with the Australia and New Zealand Clinical Trials Registry no: 12607000080426. The protocol has previously been described in greater detail [24]. Amendments to the trial protocol included analysis to determine participant's response to treatment (responders). This was necessary to meet the requirements suggested in the Osteoarthritis Research International Outcome Measures in Rheumatology (OMERACT-OARSI) guidelines [25]. In addition, the proportion of people attaining minimal clinically important improvements (MCIIs) in WOMAC physical function SF-36 domains, Visual Analogue Scale (VAS) pain (at 8 weeks) and the Timed Up & Go Test (TUG) was determined.

### OAK program

The OAK program differs from other arthritis SM programs in a number of respects. It is a disease-specific OA SM education program designed for delivery by health care professionals. Its theoretical framework uses Social Cognitive Theory [22] to enhance participants' self-efficacy and promote long-term changes in behaviour. The results of an uncontrolled quality assurance study of the OAK program were positive in terms of improvement in pain, quality of life and physical function [26]. The program was designed specifically for people with OA of the knee and for implementation in a community-based setting, thus removing the burden of health care at tertiary institutions. The education component of this program is detailed, so delivery by health care professionals is more appropriate than delivery by lay leaders. Principles and theories of SM are used to promote behavioural change. In particular, exercise and disease coping strategies are promoted within a SM construct as a means of improving quality of life and general health as well as reducing pain.

Health care professionals, including nurses, physiotherapists and occupational therapists, who deliver this program must have a minimum level of musculoskeletal education to ensure that they have the necessary knowledge and skills to present information about OA of the knee and respond accurately to complex questions. The fidelity of the OAK program is maintained by the use of a facilitators' manual with modules for program delivery each week that are designed specifically to maintain the consistency and accuracy of the information delivered.

The OAK program is conducted in a group setting with six weekly sessions of 2.5 hours each, with a baseline assessment 1 week before the start of the 6-week SM program and a second assessment the week following the completion of the program. This 8-week period is referred to as the 'clinic phase'. Attendance is voluntary; however, participants are encouraged to attend all sessions. The program is designed so that participants will progress over time by incorporating and consolidating information learned from week to week. In addition to the weekly sessions, participants are given printed information relevant to the course component discussed each week. To facilitate optimum group dynamics, the target group size is 12 participants.

The program is implemented using a holistic approach and addresses multiple aspects of care: osteoarthritis (explanation and implications), SM skills (goal-setting, problem-solving, modelling, positive thinking and improving self-efficacy), medications (types, interactions and current trends), correct use of analgesia (use, therapeutic dosing, types and side effects), pain management strategies (cognitive and pharmacologic), fitness and exercise (strength, flexibility, aerobic and balance), joint protection, nutrition and weight control, fall prevention (balance and proprioception), environmental risks, polypharmacy and coping with negative emotions. The differences between the OAK Program and the ASMP are described in Table 1.

**Table 1:** Similarities and differences between the OAK program and ASMP^a^

Comparison parameters	OAK	ASMP
Similarities		
Length of time	2.5 hours per week (6 weeks)	2.5 hours per week (6 weeks)
Group size	12 to 15	15 to 20
Setting	Community venue	Community venue
Theoretical framework	Social cognitive theory • Goal-setting (weekly) • Problem-solving • Guided imagery Cognitive behavioural therapy	Social cognitive theory • Goal-setting (weekly) • Problem-solving • Guided imagery Cognitive behavioural therapy
Small-group discussion	Occasional small groups or pairs of participants	Small groups or pairs of participants in most sessions
Program fidelity	Facilitator's manual	Facilitator's manual
Training	Leaders require training	Leaders require training
Differences		
Participants	All with OA knee	Various musculoskeletal conditions
Leaders	Health professionals (*n *= 2)	Trained leaders, one or both of whom are non-health-care professionals with arthritis themselves [58]
Osteoarthritis	Specific information on • Anatomy of knee • Pathophysiology • Disease progression • Specific treatment options • Management of OA: exercise, lifestyle, nutrition, weight loss	General overview of OA; no detailed explanations or pathophysiology Nutrition
Exercise	Actively encouraged Detailed information every session Greater emphasis on exercise Instruction and demonstration with some group participation Exercise linked to goal-setting	General information on types of exercise
Pain management	Analgesia for acute and/or chronic pain Medication Cognitive behavioural therapy	General medication overview Cognitive behavioural therapy
Medication	Specific information on • Types of medication • Current trends/alerts (such as NSAIDs) • Therapeutic dosing • Contraindications	General overview
Support group-type interaction	Occasionally	Weekly
Instruction format	Interactive Moderate didactic content Modelling Group discussion	Highly interactive Minimal didactic content Modelling Group discussion Brainstorming

^a^ASMP: Arthritis Self-Management Program; NSAID: nonsteroidal anti-inflammatory drug; OA: osteoarthritis; OAK: Osteoarthritis of the Knee Self-Management Program.

### Study design

**Figure 1:**
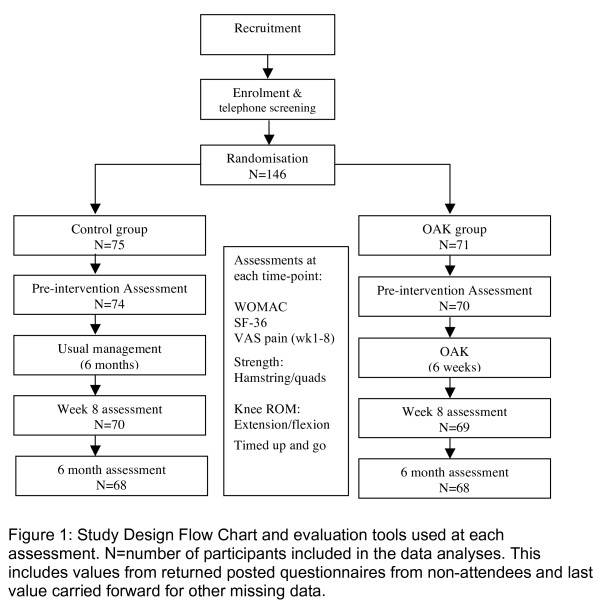
**Study design flowchart and evaluation tools used at each assessment**. The number of participants included the data analyses (N) includes values from questionnaires returned by post by nonattendees and last value carried forward for other missing data. OAK: Osteoarthritis of the Knee Self-Management Program; SF-36: Short Form 36 version 1 questionnaire; ROM: range of motion; VAS: Visual Analogue Scale; WOMAC: Western Ontario and McMaster Universities Arthritis Index.

We conducted a two-group, randomised (1:1 ratio), controlled, repeated-measures study to examine group differences regarding changes over time. Convenience sampling was employed. The research sample was selected from among people who were referred to the program. Suitable candidates were invited to enrol in the OAK program. Those who agreed to participate and provided their written informed consent were randomised either to an OAK group (immediate start) or to a control group (delayed start). For ethical reasons, those participants who were randomised to the control group were offered the opportunity to enrol in the OAK program at the conclusion of the 6-month study. Independently of the study, all participants were allowed to continue standard medical management for knee OA. Figure 1 shows the design of the study and the time points at which the outcome measures were recorded. All assessments were performed at Arthritis Western Australia.

### Participants

One hundred forty-six participants (37 male and 109 female) with established OA of the knee and mean (SD) age of 65 (8) years were enrolled into the study from primary care general practices. Inclusion and exclusion criteria are listed in Table 2. All participants who were recruited from the Perth metropolitan area and immediate surrounds provided their written informed consent prior to enrolment.

**Table 2:** Eligibility criteria^a^

Inclusion criteria	Exclusion criteria
English-speaking	Coexisting inflammatory arthritis
Age 18 years or older	Serious comorbidity
Diagnosis of OA (X-ray or clinical diagnosis)	Knee replacement scheduled in < 6 months
Referral from general practitioner or specialist	Cannot meet program time points
Able to meet program requirements	

^a^OA: osteoarthritis.

Socioeconomic status was estimated according to residential postcodes using a method developed by the Australian Bureau of Statistics: the Index of Relative Socio-Economic Disadvantage [27]. The index provides a weighted value, with a low index value representing disadvantage and a high index value representing advantage (Table 3).

**Table 3:** Characteristics of participants enrolled in an OAK program^a^

Characteristics	Control	OAK	*P *value
Mean age (SD), years	65 (8.7)	65 (7.9)	
Gender (M:F)	23:52	14:57	
Socioeconomic index by post code [27] in quintiles, *n *(%)			
Top 25%	43 (57)	46 (65)	
50% to 75%	10 (13)	9 (13)	
25% to 50%	6 (8)	8 (11)	
10% to 25%	11 (15)	6 (8)	
Bottom 10%	5 (7)	2 (3)	
Coexisting disease, *n *(%)^a^			
Total number	156	156	
Cardiovascular	48 (64)	56 (79)	
Gastrointestinal	17 (22)	21 (29)	
Musculoskeletal (other than knee OA)	32 (43)	12 (17)	
Mental health	6 (8)	7 (10)	
Endocrine	18 (24)	13 (18)	
Osteoporosis	8 (11)	8 (11)	
Other	27 (36)	39 (55)	
None	9 people (12%)	6 people (8.5%)	
Multiple coexisting diseases	49 people (65%)	43 people (60%)	
Mean (SD) incidence per person	2.39 (1.4)	2.43 (1.65)	
SF-36^b^			
Physical function	43.98 (21.2)	50.41 (22.2)	0.078
Role physical	28.38 (36.6)	40.00 (39.7)	0.070
Body pain	42.00 (19.1)	49.73 (19.0)	0.016
General health	64.81 (17.2)	65.05 (18.4)	0.936
Vitality	52.70 (21.0)	55.86 (16.4)	0.321
Social function	69.43 (26.1)	75.54 (22.1)	0.133
Role emotional	57.66 (43.1)	66.19 (42.6)	0.235
Mental health	74.92 (15.1)	75.94 (14.8)	0.683
WOMAC pain	8.00 (3.6)	6.53 (3.7)	0.020

^a^OA: osteoarthritis; OAK: Osteoarthritis of the Knee Self-Management Program; SF-36: Short Form 36 version 1 questionnaire; WOMAC: Western Ontario and McMaster Universities Arthritis Index. ^b^Percentage adds to > 100% because some participants had more than one coexisting disease. ^c^Unadjusted baseline values, mean (SD) control and OAK groups.

During the recruitment phase, the OAK program was actively promoted to general practitioners, rheumatologists and health care professionals through professional societies and to the general public through advertising and media coverage. Invitations were also extended to those people with OA of the knee who made general inquiries to Arthritis Western Australia. The OAK program and all assessments were conducted at Arthritis Western Australia, a community setting that is close to public transport and has available infrastructure to run the program and coordinate the study. This project was funded with in-kind support from Arthritis Western Australia. The research undertaken was independent from the funding body.

### Randomisation and blinding

Participants were allocated to study groups using simple randomisation performed in batches of approximately 24 depending on recruitment success. Once a group of 24 volunteers were recruited, they were randomised to either the OAK or the control group. Twenty-four premade cards (twelve interventions and twelve controls) in sealed opaque envelopes were placed in a box. An envelope was drawn from the box by an independent person to determine group allocation. Blinding of participants was not possible, owing to the nature of the intervention; however, the physiotherapists performing the assessments did not participate in the facilitation of the OAK program and thus were blind to group allocation. To maintain blinding, the physiotherapists were asked not to discuss group allocation with the participants during assessments.

### Outcome measures

The outcome measures included both primary and secondary measures. The primary measures were health status, measured using the self-administered WOMAC Osteoarthritis Index for OA of the knee (WOMAC LK3.0) [28,29]; and quality of life, measured using the SF-36 questionnaire [30,31].

The secondary measures were VAS pain [32,33] assessed at weekly intervals in the OAK group during the delivery of the knee OA program from baseline to the 8-week assessment, whereas the control group was assessed for VAS pain scores at baseline and at week 8 (see Figure 1). Functional mobility was assessed using a modified TUG test [34-36]. For this study, ascending and descending a 15-cm step was added to the outward walk. Two measurements were performed, and the average of these measurements was used for analysis. Range of motion of the knee joints was measured using a long-armed goniometer [37,38]. Isometric strength of the hamstrings and quadriceps muscles measured at 90° knee flexion using a Mecmesin force gauge dynamometer (Slinfold, UK) [39]. Parameters for each knee were measured three times. The first (practice) measurement was excluded. The two subsequent measures were averaged for analysis.

The number of responders and MCIIs achieved by participants were calculated in addition to primary and secondary outcome measures to meet the OMERACT-OARSI guidelines [25].

### Statistical power calculation

An *a priori *power calculation based on the quality-of-life outcome as measured by the SF-36 [30] was undertaken. The SF-36 was chosen because it is the least sensitive instrument and requires a greater sample size to detect changes in treatment differences with respect to pain and physical functioning in people with OA [40]. Sample size was calculated according to guidelines in the SF-36 Users Manual to determine differences in changes over time between the intervention and control groups using a repeated-measures design, allowing an intertemporal correlation of 0.60 between scores [30]. Previously, the OAK program quality assurance study SF-36 data showed an average difference of 10 points across the eight domains measured [26]. Assuming that this level of improvement was likely to be achieved in the OAK group and that no change was expected in the control group, and allowing for a 10% dropout rate, the number of participants was calculated to be 60 [30]. In the quality assurance study, the dropout rate was 5% over 3 years, so allowing for a 10% dropout rate was a conservative estimate. Differences in changes in functional ability measured using the WOMAC questionnaire, which were similar in magnitude to those previously documented [41], would also be detectable in a sample of this size.

### Data analysis

Data were analysed in a blinded manner using SPSS version 17 for Macintosh software (SPSS, Inc, Chicago, IL, USA). Treatment groups were examined for comparability at baseline. Despite randomisation, there were between group differences in severity at baseline. Therefore, baseline values (recorded in Table 3) were used as covariates in the analyses [42]. This had the effect of the preintervention mean (SEM) values' being the same at baseline in both groups. The main comparisons between groups were performed using an intention-to-treat analysis. All participants were encouraged to attend follow-up measurement sessions, regardless of their level of participation in the program. Where data were missing, the previous value was carried forward. To test the effects of treatment, between-group differences in changes over time (baseline, 8 weeks and 6 months) were examined using repeated-measures analysis of covariance. A separate analysis was conducted for each outcome variable.

For secondary analyses, a favourable response to treatment (responder) was recorded as defined by the OMERACT-OARSI criteria [25]. We used scenario D: an improvement of ≥ 50% and an absolute change of ≥ 20 points on a 100-point scale in pain or function or an improvement in at least two of the following: an improvement of ≥ 20% and an absolute change of ≥ 10 points in two of the three parameters pain, function and global health. Because patients' global health was not recorded in this study, however, only the pain and function sections of the second alternative were available.

Furthermore, the proportion of participants achieving MCIIs independently in terms of health status, quality of life, pain and the TUG test were computed for each group at each observation time point. The criteria for the MCIIs were as follows [43]:

1. Health status using WOMAC Physical Function (0 to 100)

a. Absolute change -9.1 points

b. Percentage change: -26.0%

2. VAS Pain score

a. Absolute change -1.99 points

b. Percentage change -40.8%

3. Quality of life using the SF-36 questionnaire (Body Pain and Physical Function domains)

a. Absolute change +5 points

4. TUG test

a. Percentage change -9%

The proportion of participants achieving MCIIs and the responder criteria were computed for each group at each observation time. A Pearson's χ^2 ^test not sure what the underscore is here was used to examine the effect of the treatment in terms of the proportion of MCIIs and responders. Statistical significance was inferred at a two-tailed *P *value < 0.05. The results were not adjusted for multiple comparisons, because all outcomes of interest were nominated *a priori *and such adjustment would likely have rendered all findings of interest, despite their clinical importance, nonsignificant [44].

## Results

Table 3 shows the number, characteristics and distribution of all subjects. The male-to-female ratio was not significantly different between groups (Pearson's χ^2 ^= 2.311_(1,146)_, *P *= 0.182). Sixty-eight participants from each group were assessed at 6-month follow-up examinations. All participants in the intervention group (OAK group) included in the analyses attended at least four of the six SM sessions. The mean (median) attendance in the OAK group was 5.77 (6) sessions. The reasons cited for withdrawal were overseas relocation; work, family and time commitments; and not having been randomised to the OAK group. Participants from the highest socioeconomic group were overrepresented, and approximately 90% had coexisting disease (Table 3).

### Mean differences

#### Primary measures

WOMAC Pain, Physical Function and Total scores improved more significantly in the OAK group than in the control group (Table 4). The advantage in between-group differences in changes was evident in the Physical Function and Total scores at posttreatment and 6-month follow-up; by 6 months posttreatment, however, the improvements in Pain scores were comparable between groups. There were improvements from baseline to 8 weeks in the SF-36 scales Physical Function, Role Physical, Body Pain, Vitality and Social Function in the OAK group compared with the control group. These differences were maintained at 6 months (Table 4).

**Table 4:** Results for primary outcomes based on WOMAC and SF-36: preintervention (baseline), postintervention (8 weeks) and 6 months^a^

Variables	OAK and control preintervention^b^	OAK		Control		Difference in changes between groups			
		
		8 weeks	6 months	8 weeks	6 months	Preintervention to 8 weeks	95% CI	Preintervention to 6 months	95% CI
WOMAC									
Pain^c^	7.1 (0)	5.5 (0.3)	6.1 (0.3)	7.0 (0.3)	6.7 (0.3)	-1.46	-2.18 to -0.73	-0.49	-1.26 to 0.28
Stiffness	3.6 (0)	3.1 (0.2)	3.1 (0.2)	3.6 (0.1)	3.4 (0.2)	-0.50	-0.91 to -0.08	-0.29	-0.73 to 0.15
Physical Function^c^	24.1 (0)	19.1 (0.7)	19.9 (1)	24.4 (0.7)	23.4 (0.9)	-5.55	-7.38 to -3.31	-4.35	-6.20 to -0.91
Total^c^	34.9 (0)	27.7 (1.0)	29.2 (1.2)	34.9 (1)	33.3 (1.2)	-7.23	-9.98 to -4.49	-4.08	-7.47 to -0.68
SF-36 (0 to 100)									
Physical Function^c^	48.0 (0)	54.1 (1.4)	54.2 (1.9)	48.5 (1.4)	48.5 (1.9)	5.61	1.84 to 9.37	5.67	0.40 to 10.93
Role Physical^c^	35.7 (0)	47.9 (4.0)	46.0 (4.8)	30.8 (4.0)	38.6 (4.8)	17.06	5.90 to 28.21	7.37	-5.93 to 20.67
Body Pain^c^	46.3 (0)	51.2 (1.9)	50.8 (2.1)	44.0 (1.9)	44.8 (2.2)	7.19	1.93 to 12.44	6.06	0.04 to 12.07
General Health	65.8 (0)	69.2 (1.3)	69.6 (1.7)	67.1 (1.3)	66.0 (1.7)	2.11	-1.45 to 5.67	3.59	-1.19 to 8.37
Vitality^c^	54.7 (0)	59.0 (1.5)	60.7 (1.7)	53.0 (1.5)	56.0 (1.8)	6.02	1.87 to 10.16	4.72	-0.11 to 9.55
Social Function^c^	73.8 (0)	83.0 (2.2)	77.8 (2.6)	72.3 (2.1)	72.7 (2.6)	10.72	4.81 to 16.62	4.07	-2.08 to 12.22
Role Emotional	61.7 (0)	73.7 (3.9)	70.8 (4.5)	68.5 (3.9)	69.4 (4.5)	5.18	-5.64 to 16.00	1.35	-11.06 to13.76
Mental Health	75.8 (0)	77.0 (1.3)	78.5 (1.5)	74.9 (1.3)	74.7 (1.5)	2.08	-1.42 to 5.58	3.85	-0.21 to 7.91

^a^OAK: Osteoarthritis of the Knee Self-Management Program. SF-36: Short Form 36 version 1 questionnaire; WOMAC: Western Ontario and McMaster Universities Arthritis Index. ^b^Preintervention values were the same in both groups when the baseline value was used as a covariate. Values were calculated using repeated-measures analysis of covariance with the baseline value of the dependent variable as the covariate. Data are estimated marginal means (SEM) with the mean (95% CI) differences in changes between the groups at each time point. ^c^*P *< 0.05 for baseline to 6 months.

#### Secondary measures

A trend in pain improvement that corresponded with an increase in exercise was noted in the quality assurance study. Therefore, VAS scores were assessed during the intervention phase of the study to track pain during the OAK course.

In the OAK group, VAS pain decreased 30% during the 8-week intervention phase (mean (SE) 5.21 (0.30) to 3.65 (0.29), *P *≤ 0.001), and the control group had a 17% increase in pain (5.27 (0.30) to 6.19 (0.32), *P *≤ 0.001) during the same period. The difference in the mean change between groups, from baseline to week 8, was 2.54 cm (95% CI = 1.66 to 3.41). The TUG test results showed a significant improvement in the OAK group compared with the control group postintervention and at 6 months; however, the improvement was small (Table 5) [45]. A MCII for TUG was observed in three times as many OAK group participants as control group participants at 8 weeks (OAK = 46 and control group = 15); however, this ratio was appreciably lower at 6 months (OAK = 38 and control = 26).

**Table 5:** Results for secondary outcomes: TUG, quadriceps, hamstring strength and knee joint range of motion^a^

Variable	OAK and control preintervention^b^	OAK		Control		Difference in change between groups			
		
		8 weeks	6 months	8 weeks	6 months	Preintervention to 8 weeks	95% CI	Preintervention to 6 months	95% CI
TUG (score)^c^	12 (0)	10 (0.2)	10 (0.2)	11 (0.2)	11 (0.2)	-1.3	-1.81 to -0.86	-0.72	-1.35 to -0.08
Muscle Strength (kg)									
Left quadriceps	18.9 (0)	20.3 (0.5)	19.6 (0.7)	18.6 (0.5)	18.1 (0.7)	1.65	0.34 to 2.95	1.58	-0.31 to 3.47
Right quadriceps	18.0 (0)	19.6 (0.5)	18.9 (0.7)	17.8 (0.5)	18.2 (0.7)	1.79	0.33 to 3.24	0.66	-1.37 to 2.69
Left hamstring^c^	8.0 (0)	10.1 (0.3)	9.5 (0.4)	8.6 (0.3)	8.79 (0.4)	1.47	0.63 to 2.30	0.74	-0.31 to 1.79
Right hamstring^c^	7.6 (0)	10.2 (0.3)	9.8 (0.4)	8.4 (0.3)	8.7 (0.4)	1.80	0.89 to 2.70	1.18	0.06 to 2.29
Range of motion (°)									
Left knee flexion^c^	125 (0)	126 (0.8)	126 (0.9)	123 (0.8)	123 (0.9)	2.80	0.58 to 5.02	2.26	-0.32 to 4.86
Right knee flexion	123 (0)	123 (0.9)	121 (0.9)	121 (0.9)	121 (0.9)	1.56	-0.90 to 4.02	0.02	-2.53 to 2.57
Left knee extension^c^	-4 (0)	-4 (0.3)	-4 (0.5)	-4 (0.3)	-3 (0.5)	0.1	-0.72 to 0.88	-1.39	-2.71 to -0.06
Right knee extension^c^	-4 (0)	-4 (0.3)	-5 (0.5)	-5 (0.3)	-3 (0.5)	0.9	-0.03 to 1.78	-1.18	-2.63 to 0.26

^a^OAK: Osteoarthritis of the Knee Self-Management Program; TUG: Timed Up & Go Test. ^b^Preintervention values were the same in both groups when the baseline value was used as a covariate. ^c^*P *< 0.05. Preintervention (baseline), postintervention (8 weeks) and 6-month values were calculated using repeated-measures analysis of covariance with the baseline value of the dependent variable as the covariate. Data are estimated marginal means (SEM) with the mean (95% CI) difference in changes between the groups at each time point.

Hamstring strength improved in both right and left legs in the OAK group compared with the control group. In the right hamstrings, there was a 34% improvement postintervention and a 29% improvement at 6 months. In the control group, improvements of 10% postintervention and 14% at 6 months were achieved. Similar improvements were observed in the left hamstrings (Table 5). Despite the significance of these results, they have little clinical meaning because of the limited magnitude of the improvement. There was no significant difference between groups in quadriceps strength in either the left or right legs.

Small increases in range of motion were observed. Extension in both knees and flexion of the left knee in the OAK group improved significantly compared with the control group; however, these improvements also were of questionable clinical significance because of the magnitude of the improvement.

### Responders

Following the intervention, the proportion of responders in the OAK group at 8 weeks was more than three times that in the control group (Table 6). At this posttreatment assessment, 26 people from the OAK group and 8 from the control group were classified as responders according to the prespecified criteria for response to treatment [46]. There were 22 responders in the OAK group compared to 14 in the control group at 6 months; however, the difference between groups was not statistically significant at that time point.

**Table 6:** MCII and participant responders based on changes between baseline and 8 weeks and between baseline and 6 months

			Number with MCII (%)	
			
Variables	Pearson's χ^2^	*P *values	OAK	Control
Preintervention to 8 weeks				
WOMAC Physical Function, absolute	10.84_(1, 141)_	*P *= 0.001	25 (37%)	9 (13%)
WOMAC Physical Function (%)	19.34_(1, 141)_	*P *≤ 0.001	29 (43%)	7 (10%)
SF-36 Physical Function	8.34_(1, 140)_	*P *= 0.006	40 (60%)	23 (34%)
SF-36 Pain	1.38_(1, 139)_	*P *= 0.265	23 (34%)	17 (25%)
VAS Pain, absolute	15.95_(1, 139)_	*P *≤ 0.001	27 (40%)	7 (10%)
VAS Pain (%)	17.37_(1, 139)_	*P *≤ 0.001	25 (37%)	5 (7%)
TUG	28.87_(1, 139)_	*P *≤ 0.001	46 (69%)	15 (22%)
Responders	13.59_(1, 141)_	*P *≤ 0.001	26 (39%)^b^	8 (12%)^b^
Preintervention to 6 months				
WOMAC Physical Function, absolute	3.87_(1, 135)_	*P *= 0.057	24 (14%)	14 (20%)
WOMAC Physical Function (%)	4.37_(1, 135)_	*P *= 0.043	27 (40%)	15 (22%)
SF-36 Physical Function	2.93_(1, 136)_	*P *= 0.122	40 (60%)	29 (43%)
SF-36 Pain	0.95_(1, 135)_	*P *= 0.384	31 (46%)	25 (37%)
TUG	5.10_(1, 132)_	*P *= 0.036	38 (57%)	26 (38%)
Responders	2.58_(1, 135)_	*P *= 0.123	22 (33%)^b^	14 (20%)^b^

^a^MCII: minimal clinically important improvement; OARSI: Osteoarthritis Research International; OMERACT: Outcome Measures in Rheumatology; SF-36: Short Form 36 version 1 questionnaire; TUG: Timed Up & Go Test; VAS: Visual Analogue Scale; WOMAC: Western Ontario and McMaster Universities Arthritis Index. ^b^Data are number of responders based on the OMERACT-OARSI criteria [46].

### Minimal clinically important improvements

The OAK group had a greater proportion of MCIIs on all outcome measures at all time points compared with the control group. The differences were significant for all variables apart from SF-36 Body Pain scale at 8 weeks and the SF-36 Physical Function and Body Pain scales at 6 months (Table 6). The proportion of MCIIs between the OAK and control groups was greatest immediately after intervention. In the OAK group, approximately three times as many participants were achieved a MCII compared with the control group at 8 weeks and almost twice the number at 6 months.

## Discussion

In this randomised controlled trial, we have demonstrated that participants in a SM program designed specifically for people with OA of the knee and delivered by health care professionals experienced improvements in a number of health domains that people with OAK have identified as important problems associated with their condition [47].

SM aims to motivate people to undertake the changes in behaviour necessary to improve their condition. The priorities of people with OA knee have been identified as problems with pain and activities of daily living, and their preference is to actively manage their condition [14,47]. The OAK program was designed as a community-based SM education program that aims to improve pain, function and quality of life and to empower people to address these preferences with the support of health care professionals who have expertise in this area. The OAK program incorporates education with an emphasis on OA-related information and the benefits of exercise within SM constructs to promote improved self-efficacy and changes in behaviour. Utilising the knowledge and skills of health care professionals is a chief component of the OAK program because knowledge is an important part of self-efficacy in that no amount of confidence will produce success unless the required knowledge and skills are present [48].

The mechanisms involved in successful SM are not well-understood. The highly structured nature of the intervention may be important, and other nonspecific mechanisms such as group dynamics may be contributory. Nevertheless, there appears to be consensus that the efficacy is likely to be due at least in part to increased adherence to medications [4]. This is a positive effect, especially in people with chronic diseases, for whom compliance (adherence and persistence) with all measures, including pharmacologic ones, are important in optimal management. It should be noted that in the OAK program, educational material concerning pharmacologic therapy and pain relief are included in the syllabus.

The WOMAC and SF-36 questionnaires are both tools that can demonstrate improvements in pain and in overall health status [49]; in people with OA, however, the WOMAC questionnaire is more sensitive than the SF-36 to changes in pain and physical function [40]. Improvements in pain scores demonstrated on the VAS (weeks 1 to 8) were also reflected on the WOMAC and SF-36 questionnaires in the OAK group compared with the control group, and, moreover, they were maintained to 6 months.

Similarly, in the OAK group, a significantly greater proportion of responders were observed at 8 weeks compared with the control group, though by 6 months this proportion did not differ significantly between groups. Reviews of SM show that there is a decline in the extent of improvements by 6 months. This decline is commonly observed in such studies and highlights the limited duration of the benefit that accrues due to SM in the management of OA of the knee, which, of course, requires long-term care [8,11,50]. These and other observations attest to the need for more research in this area, especially studies which investigate the differences in cost between SM programs that utilize health care professionals and layperson-led SM programs.

Determining the value that patients place on improvements in pain can be difficult. In studies in which patients were treated with nonsteroidal anti-inflammatory drugs (rofecoxib or ibuprofen), patients have perceived improvements of 9% to 10% in WOMAC scores as beneficial for OA of the knee [51]. The OAK group demonstrated better improvements in WOMAC pain of 23% between pre- and postintervention and of 13.7% from preintervention to 6 months. By contrast, at the same time points, the control group had improvements of 2.3% and 7% in WOMAC pain.

One limitation of the OAK/control study is that it compared a treatment program with a no-treatment control group. Therefore, the only blinding that could be maintained was assessor blinding, an important consequence of which is the risk of reporting, attrition and other types of bias. In addition, self-reported pain may be affected by bias, as patients are keen to 'do well' and to please health care providers by reporting an improvement when there may not have been one. Moreover, the perception of the efficacy of the treatment by the health care providers may influence how the patients perceive their pain and thus may result in an improved pain rating [52], suggesting that the bias related to no-treatment control groups is generally underestimated [53].

As with pain, there were significant improvements in quality of life and function in the OAK group compared to the control group, with improvements seen in WOMAC and SF-36 scores maintained to 6 months. Physical improvements were also maintained at 6 months compared with the control group. Self-reported functional outcome measures tend to be influenced by pain, so it is important to have functional as well as self-reported outcome measures because the combination allows a more realistic appraisal of functional ability than self-reported outcome alone [54].

The control group also demonstrated improvements in many outcomes. It is difficult to explain these improvements other than patient-provider interactions at assessments. Patients in untreated control groups may interact with health care providers. Hróbjartsson and Gøtzsche [53] suggested that the possibility of patient-provider interactions could have clinically useful effects. For example, there may have been a 'Hawthorne' effect, which is described as the awareness of being involved in a trial with resulting altered (that is, improved) behaviour or performance [55]. There was also an unexpected improvement in the number of responders in the control group from 8 weeks to 6 months. Using last value carried forward (LVCF) to replace missing data assumes that the participant's responses would have been constant from the beginning to the end of the study, and this assumption can result in the false conclusion that a difference exists when in fact there is none [56]. Using the LVCF creates the impression that the participant is in a state of equilibrium, neither better nor worse, an outcome that could be perceived as beneficial in terms of disease progression in OA because OA is a condition that generally deteriorates over time. This has the potential to bias the estimates of treatment effect [57].

Strategies to retain the study population included telephone and written notification of follow-up assessments, offers to reschedule missed assessment appointments and telephone contact to encourage rescheduling of assessments. Attendance at assessments at week 8 was 96% in the OAK group and 85% in the control group. The difference may be explained by some participants in the control group assuming that because the control period was 6 months, their participation was not required until that point. At 6 months, participation was relatively unchanged, with the OAK group's attendance being 93% and the control group's being 85%. All nonattending participants received posted self-report questionnaires with postage-paid, self-addressed envelopes enclosed. Not all participants returned the questionnaires. Although the posted questionnaires captured self-reported outcomes, they did not capture physical assessments, and other missing values were recorded with the LVCF, which is consistent with an intention-to-treat analysis.

Within-group improvements were evident on the WOMAC (Stiffness and Total scores) and the SF-36 (Physical Function, Role Physical, General Health and Vitality). The significant improvements seen in hamstring strength, but not quadriceps strength, are difficult to explain. The OAK program is not an exercise program, and, although participants are encouraged to exercise, the exercises are delivered within a SM format that requires individuals to adopt an exercise regimen that best meets their needs.

The highest socioeconomic group was overrepresented in this study. It is possible that the study results might overstate their likely impact on the wider community, because there is the potential for people with higher education levels to have better outcomes. Arthritis Western Australia is located in a middle socioeconomic area. Previous attempts to recruit from lower socioeconomic areas had limited success. Strategies for outer metropolitan and rural clinics were discussed and may be pertinent for future studies. Another possible limitation of this study is the self-initiated enrolment, which might produce potential bias because those people who volunteer may already be positively predisposed to SM [11,50].

These results reflect the improvements seen in a previously reported quality assurance study in which the OAK program was tested [26]. The use of a more rigorous study design further strengthens the earlier findings. The combined information should prove useful for planning future models of SM in arthritis care. Although the use of health care professionals as facilitators will add to the cost of such care, there is only weak evidence to support SM programs that use lay leaders. Cost analysis was not within the scope of this study. Future research comparing the OAK program with a lay leader SM program should be undertaken to determine the most effective model.

## Conclusions

In participants with OA of the knee, statistically significant improvements in pain, quality of life and function were observed in the group randomised to an OAK intervention program delivered by health care professionals compared with those randomised to a control group. The number of participants achieving MCIIs and responder criteria at 8 weeks and 6 months in the OAK group compared with the control group adds strength to these findings.

Future research is recommended to compare and estimate the cost of the OAK program in comparison to other SM models, in particular the ASMP. A comparison with ASMP would help to determine whether a policy shift between lay leaders and health care professionals is justified. Comparison of the OAK program and an exercise-alone program may provide useful information regarding the improvements noted in the OAK group. Such studies should incorporate longer follow-up assessments.

## Abbreviations

ASMP: Arthritis Self-Management Program; LVCF: last value carried forward; MCII: minimal clinically important improvement; OA: osteoarthritis; OAK: Osteoarthritis of the Knee Self-Management Program; SF-36: Short Form 36 version 1 questionnaire; SM: self-management; TUG: Timed Up & Go Test; VAS: Visual Analogue Scale; WOMAC: Western Ontario and McMaster Universities Arthritis Index.

## Competing interests

The authors declare that they have no competing interests.

## Authors' contributions

SC collected the data. SC and KB were responsible for data analysis and writing the manuscript. GC, CI, NC and JM assisted with the study design and provided comments on the drafts. All authors approved the final version of the manuscript.
